# Evaluating the Feasibility of a Telescreening Program for Retinopathy of Prematurity (ROP) in Denmark

**DOI:** 10.3390/jpm14101020

**Published:** 2024-09-24

**Authors:** Hajer A. Al-Abaiji, Regitze Bangsgaard, Mads Kofod, Carsten Faber, Ann-Cathrine Larsen, Agnes Galbo Brost, Carina Slidsborg, Kristian Klemp, Morten Breindahl, Morten Dornonville de la Cour, Line Kessel

**Affiliations:** 1 Department of Ophthalmology, Copenhagen University Hospital—Rigshospitalet, 2100 Copenhagen, Denmark; 2 Department of Clinical Medicine, University of Copenhagen, 2200 Copenhagen, Denmark; 3 Department of Neonatal and Pediatric Intensive Care—Rigshospitalet, 2100 Copenhagen, Denmark

**Keywords:** telescreening, prematurity, retinopathy of prematurity, imaging, non-physician healthcare professional

## Abstract

**Objectives**: This study investigates the feasibility of implementing telescreening for retinopathy of prematurity (ROP) using the ICON GO^®^ widefield camera operated by a non-physician healthcare professional (NPHP). We hypothesized that images captured by an NPHP are adequate to evaluate ROP changes without further examinations. Secondly, the level of agreement between independent ROP graders were evaluated based on the fundus photographs. **Methods**: National ROP screening criteria were gestational age (GA) < 32 weeks or birthweight (BW) < 1500 g. Exclusion criteria were children hospitalized and born outside the Capital Region and examinations not performed by an NPHP. The screenings were performed using the ICON GO^®^. The NPHP selected the best images for evaluation by an *on*-site ophthalmologist, regarding whether re-examination was necessary and if so, whether the re-examination was beneficial. Lastly, the images were re-evaluated by an independent *off*-site ophthalmologist. **Results**: A total of 415 screening sessions on 165 patients performed by an NPHP were included. Re-examination was necessary in three screening sessions and beneficial in two. The level of agreement between the *on*-site and *off*-site ophthalmologists regarding ROP screening outcome was *k* = 0.82, ROP stage *k* = 0.69, plus disease *k* = 0.69, and lastly ROP zone *k* = 0.37. Of the screened children, ninety-seven (58.8%) had no ROP at any time points, sixty-two (37.6%) had some stage of ROP not requiring treatment, and six (3.6%) received ROP treatment. **Conclusions**: Telemedicine screening for ROP with the ICON GO^®^ camera performed by an NPHP was feasible with an almost-perfect agreement and negligible need for re-examinations. The approach effectively identified children needing treatment, supporting the use of telescreening in ROP management.

## 1. Introduction

Retinopathy of prematurity (ROP) is a vision-threatening retinal disease seen in children born preterm with low birth weight (BW) [1,2,3]. ROP is caused by a disturbance in the development of the immature eye [4]. In Denmark, screening for ROP has been performed for many years by highly specialized ophthalmologists using binocular indirect ophthalmoscopy (BIO) [5]. Another and more recent ROP screening method is using a camera, where wide-angle retinal photographs are captured. Screening with a camera enables telescreening consultation by recording and sending electronic image files of the patient’s eyes [6]. This approach allows the patient’s condition to be assessed remotely and at a later timepoint, while simultaneously enabling comparison of disease progression or regression over time. Classification of ROP is based on the current third edition established by the International Classification of Retinopathy of Prematurity (ICROP3) [3]. The latest update of the classification was required in part due to advancements in modern technology. These advancements enable clinicians to observe more detailed retinal changes associated with ROP that need further attention.

Another advantage of telescreening for ROP is that it can be performed by non-physician healthcare professionals (NPHPs) and thus ensure a more efficient use of ophthalmological resources. In addition, screening with a camera may be less stressful for the children compared to BIO and the images can be stored and reviewed to ensure patient safety and enable quality studies and further research [7]. BIO is, however, still considered the gold standard of ROP screening [8,9]. A recently published study, which presented the latest advancements in ROP telemedicine, concluded that telemedicine can be an alternative to BIO [10]. However, it is important to note that there are huge differences in both the incidence and severity of ROP depending on the population studied. Furthermore, ROP telemedicine studies have employed a variety of designs (retrospective, prospective), methods (camera model, BIO as reference, digital BIO), professional performing the exam (ophthalmologist, non-physician), grader (ophthalmologist, non-physician, or AI), and outcome measures (severe ROP, presence of plus disease, ROP classification), where most have focused on the presence of plus disease [9,11,12,13,14,15,16,17].

Several original studies and a review have already compared screening outcomes between widefield cameras versus BIO [9,11,18,19]. This study focuses on comparing evaluations from independent ophthalmologists of images captured by an NPHP using exclusively a widefield camera.

The purpose of this study was to evaluate whether an NPHP can achieve reliable image quality in a new telemedicine setup for ROP in the Capital region of Denmark. We analyzed the frequency of re-examinations by an *on*-site ophthalmologist due to insufficient image quality. Additionally, we evaluated the level of agreement between independent ophthalmologists regarding ROP stage, zone, plus disease, and screening outcome.

## 2. Materials and Methods

### 2.1. Study Population and Recruitment

At the beginning of the study, the Danish national screening criteria for ROP was gestational age (GA) < 32 weeks or birthweight (BW) < 1500 g. Since April 2023, the national guidelines narrowed to GA < 32 weeks and BW < 1600 g. Screening starts no later than 5 weeks postnatally and at a post-menstrual age (PMA) of at least 31 weeks. The children were recruited as part of the clinical routine by healthcare staff involved in screening for ROP.

Children included in our study were born and hospitalized at one of the four neonatal intensive care units (NICUs) within the Capital region of Denmark. Children were included in the study in a one-year period from 5 September 2022 to 25 September 2023. The screening sessions included were performed by the NPHP using the ICON GO^®^ camera (Phoenix ICON™ trademark of Neolight, LLC, Phoenix, AZ, USA). Exclusion criteria encompassed screening sessions performed using other methods than the ICON GO^®^ or sessions conducted by an ophthalmologist on days when the NPHP was physically absent, such as during holidays or work leave. These exclusions were solely due to the absence of the NPHP, and not due to difficult cases or an inability to perform the screenings. The general condition of the child was always discussed with the team managing the child prior to the ROP screening to ensure that the child was fit for the examination. The ROP screening was postponed in cases where the neonatologists evaluated that the child was too weak, and if the *on*-site ophthalmologist could accept the level of risk by postponing the screening. The NPHP performed the screening sessions three days a week, with one of three *on*-site ophthalmologists available each day.

Fundus pigmentation was noted at the first screening session due to its potential impact on image processing and evaluation. Fundus pigmentation was categorized as defined by the American Academy of Ophthalmology as follows: light (choroidal vessels visible within the macula), medium (choroidal vessels visible outside the arcades, but not within the macula), or dark (choroidal vessels not visible within the macula nor outside the arcades) [20].

### 2.2. Preparation of the Child Prior to ROP Examination

Local anesthesia was obtained using one drop of 0.4% oxybuprocaine, followed by two instillations with 10 min intervals of either 0.5% tropicamide and 2.5% phenylephrine or cyclomydrile (cyclopentolat hydrochlorid 0.2%/phenylephrin hydrochlorid 1%) applied by the neonatal nurse. After 30–45 min, the eyes were expected to be fully dilated. In cases of minimal effect, the eye drops were re-administered. Prior to inserting the eye speculum, local anesthesia was re-administered. Viscotears (Bausch & Lomb Nordic AB, Stockholm, Sweden) eye gel (2 mg/g carbomer) was administered for two main purposes: to lubricate and protect the cornea, and to serve as a bridge between the camera tip and the cornea.

To minimize the burden of the screening examinations, the children were swaddled and offered sugar water or breast milk, along with a pacifier, to help them stay calm and distracted. Vital signs of the child were monitored by the neonatal nurse assisting with the examination and screening was paused depending on the condition of the child.

### 2.3. Telescreening Setup

Before the study began, the NPHP completed five months of hands-on training under the supervision of an ROP expert (ophthalmologist). Additionally, the NPHP underwent retinal imaging training conducted by a clinical education specialist, who also reviewed images captured by the NPHP and who could approve the NPHP as a certified imaging technician. The clinical education specialist was from Neolight, Scottsdale, AZ, USA, which is a medical technology company providing solutions for neonatal care, for instance the photography device for ROP imaging as the ICON GO^®^ (www.theneolight.com). The *on*- and *off*-site graders completed a case-based online training in grading ROP at the home page of the American Academy of Ophthalmology [21].

A portable wide-angled ICON GO^®^ camera (Phoenix ICON™ trademark of Neolight, LLC, Phoenix, AZ, USA) was used for obtaining fundus photographs. Prior to each screening session, the ICON GO^®^ was re-calibrated and the automated white balance function was set using a standard white background to ensure the level of brightness was equal to white during the recordings. The bedside setup consisted of an NPHP who was in the room with the child and a neonatal nurse. The dilating effect of the eyedrops was evaluated prior to the screening, and re-dilating drops were given if the pupil of either eye was < 6 mm in diameter. The NPHP recorded a video of the fundus of each eye. The initial video duration was set to a maximum of 90 s per eye. The examination was terminated either when the NPHP was satisfied with the images or when further technical improvement was not possible. From the video, the best frames per eye presenting all four quadrants and the central retina were chosen. The images were digitally exported to the Topcon IMAGEnet i-base V3.23.0 database (Topcon Europe Medical B.V., Capelle Aan De Ijssel, The Netherlands).

The *on*-site ophthalmologist was available at the same time and hospital where the screenings were performed, and reviewed the images outside the screening room. The *on*-site ophthalmologist decided if the image quality was sufficient to plan the screening outcome. The *on*-site ophthalmologist informed the parents on the screening results and outcome. In cases of insufficient images, the *on*-site ophthalmologist decided if the child should be re-examined and by which method (ICON GO^®^ or BIO) to ensure patient safety. All images obtained during the screening session were re-evaluated by an independent *off*-site ophthalmologist within 3 days of the screening session. The *off*-site ophthalmologist worked from a separate office and did not have any direct contact with the patients. The *on*-site and *off*-site ophthalmologist were blinded to each other´s assessment. In cases of disagreement, the *off*-site ophthalmologist´s decision would overrule the *on*-site ophthalmologist. The *off*-site ophthalmologist was also the surgeon who would make the final decision regarding Type 1 ROP treatment. There are no official guidelines regarding treatment for late ROP sequelae, hence these cases were discussed in case of disagreement. Information regarding the child’s GA, BW, and PMA on the screening day was available for all ophthalmologists during the image evaluation, as were previously captured images in order to evaluate a possible regression or progression in ROP.

### 2.4. ROP Classification and Screening Outcome

ROP classification, including stage, zone, and plus disease, was assessed by the ophthalmologists for each eye of every patient according to the third International Classification of ROP [3]. All ophthalmologists used a standard guideline to determine the screening outcome based on the overall ROP evaluation of both eyes, as shown in Table 1. If one eye showed more severe clinical findings, the screening outcome was based on that eye. The ROP treatment criteria for Type 1 were based on the randomized clinical trial titled the Early Treatment for Retinopathy of Prematurity Study (ETROP) [22].

### 2.5. Data Access and Management

The children’s medical files were reviewed prior to each screening session to track where the child was hospitalized and to achieve a status of the general health condition of the child. Information on GA, BW, and PMA was extracted from the medical file. PMA and GA were converted from total days to weeks.

### 2.6. Statistical Methods

Descriptive statistics for GA, BW, and PMA were tested for normality. Data that were non-parametric or had a small sample size were analyzed using the Mann–Whitney test to assess differences between included and excluded patients. Normally distributed variables were analyzed using the unpaired *t*-test.

The screening outcome was defined as the final conclusion for each session, which included an overall evaluation of both eyes. However, the ROP classification was assessed separately for each eye of every patient by both on-site and off-site ophthalmologists. To simplify the results section, we tested for statistical differences between the eyes. If no significant differences were found between the right and left eyes, we would present the ROP classification for one eye only. Consequently, the kappa statistics were provided for the screening outcome (based on both eyes) and the ROP classification (based on one eye).

The level of agreement regarding the screening outcome and ROP classification (zone, stage, and presence of plus disease) was analyzed by Cohen’s kappa. The weighted kappa was presented for each of the three *on*-site ophthalmologists compared with the *off*-site ophthalmologist, as well as for all *on*-site ophthalmologists collectively compared with the *off*-site ophthalmologist. The kappa statistic has a range of k < 0.00 to k = 1, sorted in six categories depending on the value. The strength of the agreement according to the kappa value is graded as poor (k < 0.00), slight (k = 0.00–0.20), fair (k = 0.21–0.40), moderate (k = 0.41–0.60), substantial k = 0.61–0.80), and almost perfect (k = 0.81–1.00) [25]. The *p*-values computed as part of the kappa statistics were not presented, because the assumption of no association was unreasonable [26] (pp. 433–437).

The Chi-square test was performed to analyze the statistical differences between the right and left eyes in the ROP classification (stage, zone, and plus disease) for each of the *on*-site and *off*-site graders. A *p*-value less than 0.05 was considered statistically significant. Data were collected in REDcap (Research Electronic Data Capture, Vanderbilt University, Nashville, TN, USA) and analyzed using Stata 18.5 (StataCorp LLC, College Station, TX, USA).

### 2.7. Approvals

The Danish Data Protection Agency approved the study (P-2020–1206). Ethical board approval was waived by The Danish National Medical Research Ethics Committee (VMK, decision number VMK-protokol_200921_V1). The legal department at Rigshospitalet decided that the study could be performed by approval from the local head of department.

## 3. Results

### 3.1. Study Population Characteristics

**Figure 1:**
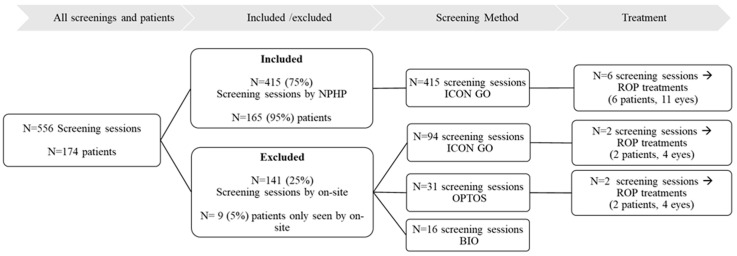
Flowchart of the included patients and screenings during study period. Nine patients received treatment on both eyes, and one patient was treated on the left eye only during the study period. Abbreviations: non-physician healthcare professional (NPHP), binocular indirect ophthalmoscopy (BIO), and retinopathy of prematurity (ROP).

A total of 556 screening sessions were performed on 174 patients during the study period. The 141 (25%) screening sessions exclusively performed by an ophthalmologist were excluded from the study. Thus, the study was based on 415 screening sessions (75% of all performed screening sessions) performed by the NPHP on 165 children (53% males). Additionally, nine (5%) patients were exclusively examined by the on-site ophthalmologists, see Figure 1. We examined the data for inclusion bias and found that the excluded patients compared to the included had a significantly higher mean GA (30.9 ± 0.7 and 28.9 ± 2.4 weeks, *p* = 0.017), mean BW (1442.6 ± 176.7 and 1206.9 ± 394.3 g, *p* = 0.04), and mean PMA at the time of screening (38.01 ± 4.4 and 36.2 ± 2.6 weeks, *p* = 0.0003), respectively.

**Figure 2:**
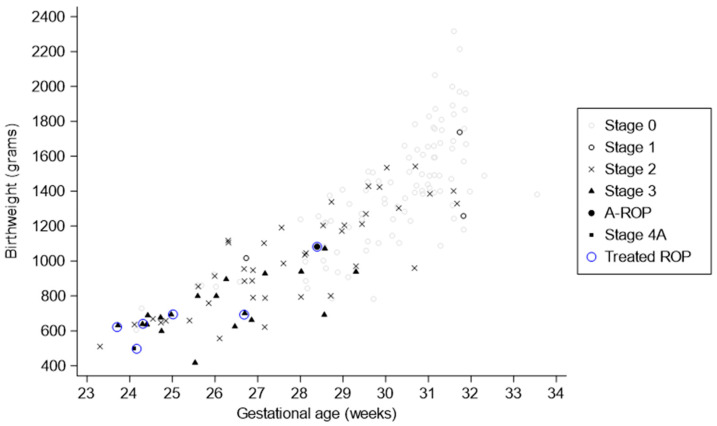
Scatterplot (jitter) of birthweight and gestational age for each patient differentiating the maximum stage of retinopathy of prematurity (ROP) detected during the screening period. Patients receiving treatment for ROP are marked with a blue open circle; the symbol inside the open circle is for the same patient.

Each child was on average screened 2.5 times by the NPHP during the screening period, ranging from 1 to 12 screening sessions per patient. A total of 97 (58.8%) patients did not develop any ROP (Stage 0), whereas *n* = 68 (41.2%) were graded as having ROP (Stage 1–4A) during the screening period. ROP stage, using the most severe stage encountered for each patient during the screening period, was Stage 1: *n* = 3 (1.8%), Stage 2: *n* = 42 (25.5%), Stage 3: *n* = 21 (12.7%), A-ROP: *n* = 1 (0.6%), Stage 4A: *n* = 1 (0.6%), see Figure 2. The patient evaluated as having ROP Stage 4A was examined using ultrasound in full anesthesia initially before treatment. Here, the surgeon found Stage 3 ROP in all four quadrants but retinal detachment was not observed. No patients were graded as having Stage 4B or 5.

Six patients (3.6%) (eleven eyes) included in the study received laser therapy for ROP, see Figure 2. The median PMA at the time of treatment was 36.3 weeks (IQR: 34.9–42.7, range: 33.6–46.7), see Table 2. The decision to treat was based on screening sessions performed by the NPHP, see Figure 1. Four out of six cases were due to Type 1 ROP and A-ROP. The remaining two cases received treatment due to structural changes that did not meet the treatment criteria for Type 1 ROP (e.g., suspicion of retinal traction or fold with or without the presence of hemorrhages or persistent Stage 3 ROP), see Table 3.

One patient received treatment on one eye only. Five patients were treated on both eyes; in one of those eyes, the last treatment was performed outside the study period at a PMA of 41 + 4. Additionally, one patient had a maximum Stage 0 seen in Zone II without plus during the study period but received treatment after the end of the study due to progression to post Zone II Stage 3 with plus disease. The screening sessions during the study period but prior to the development of treatment warranting ROP are included in the kappa analyses.

The majority *n* = 107 (65%) had a lightly pigmented fundus, followed by 42 (25%) with a medium pigmented fundus, and lastly 16 (10%) had a dark fundus. Forty-five children (27%) were twins (twenty-two twin pairs, where one twin was excluded because screening was performed only by the on-site ophthalmologist).

### 3.2. Correlation between Image Quality, Satisfaction, and Fundus Pigmentation

In three (0.7%) screening sessions, the on-site ophthalmologist deemed the images unsatisfactory, while the NPHP had only flagged two of these sessions as unsatisfactory. In the first case, BIO was performed by the on-site ophthalmologist enabling visualization of the ridge, while the NPHP was similarly unsatisfied due to technical difficulties in imaging a darkly pigmented retina. In the second case, the on-site ophthalmologist was unsure whether there was a hemorrhage or an artifact on the images and performed BIO to rule out hemorrhages, while the NPHP was likewise not satisfied with the visualization of Zone II. In the last case, the on-site ophthalmologist supplemented the images with BIO but did not obtain new information; comparatively, the NPHP was satisfied with the image quality.

The off-site ophthalmologist was unsatisfied with two (0.5%) screening sessions. In one case, not all the images had been correctly uploaded to the imaging program; in another case, the ridge was not sufficiently visualized. The screening sessions deemed unsatisfactory by the on-site and off-site ophthalmologists did not overlap. The likelihood of deeming images unsatisfactory increased with greater retinal pigmentation for both the NPHP (*p* = 0.0001) and the on-site ophthalmologist (*p* = 0.014). However, this trend was not observed for the off-site ophthalmologist (*p* = 0.26).

### 3.3. Agreement on Screening Outcome between On-Site and Off-Site Ophthalmologists

The outcome of the screening session could be to treat ROP, screen again in ≤1 week, 1–2 weeks, 2 weeks, 2–3 weeks, or terminate the screening, based on the worst eye of each patient. We evaluated the level of agreement for the screening outcome between three on-site and one off-site ophthalmologists and found an ‘almost-perfect’ agreement between all ophthalmologists (k = 0.82, weighted kappa). The screening outcome deviated by one level in sixty-seven (16%) screening sessions, two levels in twenty (5%), and three levels in two (0.5%) screening sessions, see Table 4. No screening outcomes deviated by four or more levels. Although the frequency of disagreement regarding screening outcomes was slightly higher in darkly pigmented retinas, it was not significant. Overall, the frequency of agreement and disagreement did not vary significantly across the different pigmentation levels of light (79.2% and 20.8%), medium (79.3% and 20.7%), or dark (74% and 26%), respectively (*p* = 0.69).

### 3.4. Agreement on ROP Classification

Stage, zone, and plus disease did not differ significantly between the right and left eyes for each of the on- and off-site graders (*p* > 0.05). Data in this section are therefore presented for the right eye only.

Stage was categorized as Stage 0, Stage 1, Stage 2, Stage 3, A-ROP, and regression ROP. The level of agreement for ROP stage between the off-site and three on-site ophthalmologists was substantial (weighted kappa k = 0.69). ROP stage deviated by one level in twenty-eight (6%) screening sessions, two levels in twenty-three (5%), three levels in twenty-one (5%), four levels in two (0.5%), and by five levels in the last fifteen (3%) screening sessions, see Table 5.

Zone was categorized as Zone I by notch, Zone I, posterior Zone II, Zone II, and Zone III. The weighted kappa between the one off-site and three on-site ophthalmologists was k = 0.37, equivalent to a fair agreement. The frequency of deviations by one level was *n* = 92 (22%) and by two levels was *n* = 1 (0.2%), see Table 6.

Plus disease was categorized as no plus, pre-plus, and plus. The weighted kappa regarding the presence of plus disease for the right eye was k = 0.69, corresponding to a substantial level of agreement. Deviations by one level were *n* = 24 (6%) on the right eyes, and *n* = 25 (6%) on the left eyes, when comparing all three ophthalmologists, see Table 7. No deviations by two levels occurred (plus versus no plus).

## 4. Discussion

### 4.1. Main Findings

In this paper, we investigated the feasibility of an NPHP performing ROP screening on prematurely born babies using wide-angle fundus photographs obtained by the ICON GO^®^ camera. Images were evaluated independently by three *on*-site and one *off*-site ophthalmologist. The *on*-site ophthalmologist was present outside the screening room to ensure the safety of the study in cases where screening by the NPHP was deemed insufficient to reach a decision on the screening session outcome. Re-screening by the *on*-site ophthalmologist was performed in three out of four hundred and fifteen screening sessions and in two out of three, the re-examination was beneficial for reaching a decision. Photographs were re-evaluated by an *off*-site ophthalmologist who also had the final decision for the treatment of Type 1 ROP. There was an ‘almost-perfect’ level of agreement between the *on*-site and *off*-site ophthalmologists regarding the screening outcome, assessed by Cohen’s kappa, followed by ROP stage and presence of plus disease, and lastly by ROP Zone in a descending order.

### 4.2. Defining Good Image Quality

High-quality images are important in order to classify ROP and decide the screening outcome. In our study, the certified NPHP was focused on image quality, including brightness, focus, and visualization of the retinal periphery. The degree of pigmentation of the ocular background played a significant role in the ease of obtaining images of sufficient quality as observed elsewhere [20]. The ophthalmologists had the possibility to improve the image brightness and contrast in the viewing software which was unavailable to the NPHP during the screening session. This indicates that slightly lower quality images may be still useful to make an adequate assessment, which supports the low rate of re-examinations observed in our study [12]. It is crucial to establish a standardized training program for NPHPs who operate widefield cameras during these screenings. The e-ROP study developed a thorough training program that included guidelines for imaging, selecting, and exporting retinal images [27]. This program can be adapted as a model for ROP telescreening, helping to ensure consistent and accurate results.

### 4.3. Potential Inclusion Bias

Our analysis indicated that the excluded patients were older than the included patients. These findings support the unequal distribution of ROP zone between the groups, with a higher frequency of Zone III involvement in the excluded screening sessions. These results were enhanced by the superior ability of examining Zone III with BIO compared to the camera. In summary, patients at high risk of acute ROP were primarily examined by the NPHP and the study results might overrepresent the most severe cases. Thus, considering the aim of the project, evaluating the feasibility of ROP telescreening performed with the ICON GO^®^, we do not believe that potential inclusion bias has a negative impact on the clinical implications of our findings.

### 4.4. Discrepancies in ROP Classification between Ophthalmologists

This is the first Danish study to analyze the feasibility and setup of a telescreening program in detecting ROP performed by an NPHP. Studies have been conducted elsewhere with non-physicians as the technicians [11,12,13,15,28,29]. These studies have shown promising results with some degree of variability in ROP classification, but a good level of agreement regarding ROP requiring treatment. The study by Campbell et al. presented the total percentage of image-based discrepancies between two experts for zone, stage, and plus disease, 8%, 40%, and 18%, respectively [15]. In comparison, our study showed discrepancies of 22.5% for zone, 19.5% for stage, and 5.7% for plus disease. The inconsistency between the studies can be explained by the fact that more categories were included in our study. For instance, the zone was categorized in I, II, and III, whereas we additionally had Zone I by notch and posterior Zone II. The agreement on defining the ROP zone was notably inconsistent in our study. Zone I is defined as a circular area centered on the optic nerve, with a radius equal to twice the distance from the macula to the optic disc center [3]. This definition does not account for the ocular growth during the neonatal period. A study aimed at predicting the Zone I area found that changes in axial length had the most significant impact [30]. This finding may help explain the discrepancies in ROP zone evaluations. Adopting a standardized template for defining Zone I based on the PMA of the child could potentially improve consistency in future research.

A previous Danish study investigated the inter-rater agreement regarding particularly plus disease. The available fundus photographs captured prior to ROP treatment were evaluated retrospectively by four experts. The level of agreement was inadequate for plus disease; however, a good agreement regarding treatment was presented, comparable to our study [31]. The variability in grading plus disease is concerning as it can determine whether a child receives treatment. This may lead experts to over-diagnose plus disease to meet the treatment criteria for Type 1. Conversely, if the camera applies pressure to the eye during imaging, the retinal vessels might misleadingly appear with less severe plus disease [19].

The level of agreement regarding ROP outcome analyzed with kappa statistics in this study was ‘almost-perfect’. During our study, six patients fulfilled our criteria for receiving treatment, of whom three were due to Type 1 ROP and one due to A-ROP. In these cases, there was complete agreement regarding the indication for treatment. Lastly, two had late structural ROP complications, and disagreements regarding treatment occurred. Late structural changes due to ROP seen in children today are not described as part of the recommendations from the ETROP study conducted in 2004 and therefore disagreements among the graders were not surprising [22]. Treatment for ROP, outside the current recommendations, have been reported and are often related to Type 1 in the fellow eye, logistical considerations, Stage 3 with pre-plus, and concerning late structural changes due to ROP [32,33]. Further, these patients often have a PMA > 40 weeks, which also was the case in our study. It is nonetheless reassuring that our telescreening setup showed a 100% feasibility within the current treatment recommendations and additionally managed to represent these cases.

### 4.5. Strengths and Limitations

We evaluated the feasibility of ROP screening by an NPHP but did not compare to BIO performed on the same patients. We decided against performing both fundus photography and BIO due to the vulnerable condition of the children as this would significantly increase the duration of the examination. Some challenges must be addressed in using widefield cameras in screening for ROP. Firstly, it may prolong the examination time when the NPHP is striving for the best frames to be evaluated by the ophthalmologist compared to BIO performed by an experienced ophthalmologist. Additionally, the patient’s family must wait for the ophthalmologist to review the images and cannot be informed about the screening outcome immediately after the screening session. Lastly, a thorough education of healthcare professionals in ROP screening with a widefield camera is essential to ensure patient safety. However, these challenges are deemed manageable, and lessons learned from other telescreening setups could be applied. So far, wide-angled cameras have been evaluated as a reliable supplement for BIO, the gold-standard within ROP screening, but has not yet replaced it [9,28]. With the exponential development of technology, a potential benefit of including artificial intelligence in telescreening for ROP has already been investigated and would expectedly support decision-making in ROP in the nearest future [34]. Telescreening in ROP would be a benefit due to optimized resources within staff, evaluation of ROP in a stressless environment, avoiding unnecessary transfer of preterm babies and their families between hospitals for evaluation, and the possibility of tracking regression and progression in ROP.

Despite a high number of included screening sessions in this study, the number of patients receiving ROP treatment was low. The strength of this study is that all ROP treatments were performed in the same center in Denmark; accordingly, all patients fulfilling our treatment criteria for Type 1 ROP were included.

## 5. Conclusions

Telescreening with the ICON GO^®^ camera performed by an NPHP showed an ‘almost-perfect’ agreement regarding screening outcome and the need for re-examinations was negligible. A standardized education and certification for imaging ROP should be setup for healthcare professionals to ensure patient safety. This setup can open up possibilities for implementing telemedicine on a national level in Denmark.

## Figures and Tables

**Table 1:** Screening outcome depending on ROP classification.

Screening	Treatment
One-week-or-less Follow-up	Type 1 ROP
Zone I: immature vascularization, no ROP Zone I: Stage 1 or Stage 2 ROP Immature retina extending into posterior Zone II Suspected presence of AP-ROP Stage 3 ROP	Zone I with plus disease Zone I Stage 3 without plus Zone II Stage 2 or 3 with plus disease
One- to two-week Follow-up	A-ROP
Posterior Zone II: immature vascularization Zone II: Stage 2 ROP Zone I: unequivocally regressing ROP	A-ROP is characterized by the rapid onset of abnormal neovascularization and severe plus disease, occurring without the chronologic stage progression of ROP
Two-week Follow-up	Hybrid-ROP
Zone II: Stage 1 ROP Zone II: no ROP, immature vascularization Zone II: unequivocally regressing ROP	Stage 3 including A-ROP characteristics in same eye with plus disease
Two- to three-week Follow-up	
Zone III: Stage 1 or 2 ROP Zone III: regressing ROP	
Stop Screening	
PMA > 36 weeks AND no sign of ROP in earlier screenings in Zone I or entire Zone II	
Fully regressed ROP	

Note: Screening and treatment schedule inspired by Fierson et al. (2018) [23]. ROP classification following the current guidelines as defined by ICROP3 [3]. The definition of Hybrid-ROP is as described by Sanghi et al., 2012 [24].

**Table 2:** PMA (weeks) at maximum ROP stage during the study period.

	N (%)	PMA, Median	PMA, (Min–Max)
Any ROP	68 (41.2)	34.6	(31.3–46.7)
Stage 1	3 (1.8)	36.7	(32.1–36.7)
Stage 2	42 (25.5)	34.1	(31.3–39.4)
Stage 3	20 (12.7)	34.9	(31.9–46.7)
A-ROP	1 (0.6)	34.9	(34.9–34.9)
Stage 4A	1 (0.6)	42.7	(42.7–42.7)
Treatment for ROP	6 (3.6)	36.3	(33.6–46.7)

Note: Post-menstrual age (PMA) in weeks tabulated with the maximum stage of retinopathy of prematurity (ROP) during the screening period. No children had re-activated ROP or Stage 4B or 5. PMA at the first treatment for ROP is also presented. Patients who did not develop ROP during the study (*n* = 97, 58.8%) are not presented in this table.

**Table 3:** Specifications of patients who received treatment for ROP.

ID	GA	BW	ROP Classification and Outcome	Indication	PMA	Weight
166	23 + 5	628	***On*-site** LE: Post. Zone II. Stage 3. Plus Plan: Treatment LE ***Off*-site** LE: Post. Zone II. Stage 3. Plus Plan: Treatment LE	Type 1	36 + 4	2505
172	26 + 5	698	***On*-site** RE: Zone II. Stage 3. Pre-plus LE: Zone II. Stage 3. Pre-plus Plan: Treatment BE ***Off*-site** RE: Post. Zone II. Stage 3. Pre-plus LE: Post. Zone II. Stage 3. Plus Plan: Treatment BE	Type 1	36 + 2	2030
173	25 + 0	698	***On*-site** RE: Zone II. Stage 3. Plus LE: Zone II. Stage 3. Plus Plan: Treatment BE ***Off*-site** RE: Post. Zone II. Stage 3. Plus LE: Post. Zone II. Stage 3. Plus Plan: Treatment BE	Type 1	33 + 5	2080
12	28 + 3	1082	***On*-site** RE: Zone I by notch A-ROP. No plus Plan: Treatment RE ***Off*-site** RE: Zone I by notch A-ROP. No plus Plan: Treatment RE	Other Rapid ROP progression with hemorrhages	35 + 0	1915
66	24 + 1	500	***On*-site** RE: Zone II. Stage 3. Pre-plus LE: Zone II. Stage 4A. Pre-plus Plan: Treatment BE ***Off*-site** RE: Zone II. Stage 3. Pre-plus LE: Zone II. Stage 3. Pre-plus Plan: Screening ≤ 1 week	Other worrisome structural ROP changes	42 + 6	3256
127	24 + 2	640	***On*-site** RE: Zone II. Stage 3. Pre-plus LE: Zone II. Stage 3. Pre-plus Plan: Screening ≤ 1 week ***Off*-site** RE: Post Zone II. Stage 3. Hybrid ROP Pre-plus LE: Zone II. Stage 3. Pre-plus Plan: Treatment BE	Other worrisome structural ROP changes	47 + 5	4100

Note: This table summarizes the patients, screened by the non-physician healthcare professional (NPHP), who received laser therapy treatment for ROP as the first treatment. Abbreviations: right eye (RE), left eye (LE), both eyes (BE), gestational age (GA), birth weight (BW), retinopathy of prematurity (ROP), and post-menstrual age (PMA).

**Table 4:** Level of agreement for screening outcome based on the most affected eye if unequal.

				*Off*-Site				
		**Treat**	**≤1 w**	**1–2 w**	**2 w**	**2–3 w**	**Terminate**	**Total**
***On*-site** **1**	**Treat**	4	1	0	0	0	0	5
	**≤1 w**	0	33	5	0	0	0	38
	**1–2 w**	0	3	37	4	0	1	45
	**2 w**	0	0	11	33	0	4	48
	**2–3 w**	0	0	1	1	0	0	2
	**Terminate**	0	0	1	2	0	25	28
	**Total**	4	37	55	40	0	30	166
***On*-site** **2**	**Treat**	0	0	0	0	0	0	0
	**≤1 w**	0	4	8	0	0	0	12
	**1–2 w**	0	0	27	5	0	0	32
	**2 w**	0	0	6	41	0	4	51
	**2–3 w**	0	0	1	0	0	3	4
	**Terminate**	0	0	0	2	0	49	51
	**Total**	0	4	42	48	0	56	150
***On*-site** **3**	**Treat**	0	0	0	0	0	0	0
	**≤1 w**	1	12	5	0	0	0	18
	**1–2 w**	0	0	19	1	0	0	20
	**2 w**	0	0	5	16	0	1	22
	**2–3 w**	0	0	2	6	2	2	12
	**Terminate**	0	0	0	3	0	24	27
	**Total**	1	12	31	26	2	27	99
***On*-site** **all**	**Treat**	4	1	0	0	0	0	5
	**≤1 w**	1	49	18	0	0	0	68
	**1–2 w**	0	3	83	10	0	1	97
	**2 w**	0	0	22	90	0	9	121
	**2–3 w**	0	0	4	7	2	5	18
	**Terminate**	0	0	1	7	0	98	106
	**Total**	5	53	128	114	2	113	415
	Agreement	Expected agreement			Kappa, wgt (95% CI)		SE	
*On*-site 1 × *off*-site	94.58%	69.79%			0.82 (0.73–0.88)		0.05	
*On*-site 2 × *off*-site	94.00%	63.80%			0.83 (0.79–0.86)		0.06	
*On*-site 3 × *off*-site	93.54%	67.69%			0.80 (0.77–0.84)		0.07	
ALL × *off*-site	94.55%	68.40%			**0.82** (**0.82–0.85**)		0.04	

Note: Screening outcome tabulated between the *on*-site and *off*-site ophthalmologists’ evaluation based on either eye with the most severe retinopathy of prematurity (ROP). The highlighted gray cells are decisions with full agreement. Abbreviations: week (w), weighted (wgt), confidence interval (CI), and standard error (SE).

**Table 5:** Level of agreement of ROP stage—Right eye.

				*Off*-Site				
		**Stage 0**	**Stage 1**	**Stage 2**	**Stage 3**	**A-ROP**	**Reg ROP**	**Total**
***On*-site** **1**	**Stage 0**	69	1	3	1	0	1	75
	**Stage 1**	0	1	0	0	0	0	1
	**Stage 2**	5	2	32	5	0	0	44
	**Stage 3**	1	0	5	21	0	2	29
	**A-ROP**	0	0	0	0	1	0	1
	**Reg ROP**	0	1	5	0	0	9	15
	**Total**	75	5	45	27	1	12	165
***On*-site** **2**	**Stage 0**	100	0	2	0	0	3	105
	**Stage 1**	2	1	0	0	0	0	3
	**Stage 2**	8	1	19	0	0	1	29
	**Stage 3**	0	0	3	0	0	0	3
	**A-ROP**	0	0	0	0	0	0	0
	**Reg ROP**	3	0	5	0	0	2	10
	**Total**	113	2	29	0	0	6	150
***On*-site** **3**	**Stage 0**	43	0	0	0	0	0	43
	**Stage 1**	0	2	0	0	0	0	2
	**Stage 2**	1	0	20	2	0	1	24
	**Stage 3**	0	0	4	9	0	0	13
	**A-ROP**	0	0	0	0	0	0	0
	**Reg ROP**	7	1	5	0	0	4	17
	**Total**	51	3	29	11	0	5	99
***On*-site** **all**	**Stage 0**	212	1	5	1	0	4	223
	**Stage 1**	2	4	0	0	0	0	6
	**Stage 2**	14	3	71	7	0	2	97
	**Stage 3**	1	0	12	30	0	2	45
	**A-ROP**	0	0	0	0	1	0	1
	**Reg ROP**	10	2	15	0	0	15	42
	**Total**	239	10	103	38	1	23	414
	Agreement	Expected agreement		Kappa, wgt (95% CI)		SE		
*On*-site 1 × *off*-site	92.36%	66.23%		0.77 (0.77–0.83)		0.06		
*On*-site 2 × *off*-site	89.67%	74.94%		0.59 (0.52–0.61)		0.07		
*On*-site 3 × *off*-site	87.12%	60.56%		0.67 (0.65–0.74)		0.07		
ALL × *off*-site	90.24%	68.45%		**0.69 (0.64–0.79)**		0.04		

Note: Retinopathy of prematurity (ROP) stage tabulated between the on-site and off-site ophthalmologists for the right eyes. Stage evaluations with full agreement are marked in grey. Abbreviations: aggressive ROP (A-ROP), regressed ROP (reg ROP), weighted (wgt), confidence interval (CI), and standard error (SE).

**Table 6:** Level of agreement of ROP Zone—Right eye.

				*Off*-Site			
		**Zone I Notch**	**Zone I**	**Post Zone II**	**Zone II**	**Zone III**	**Total**
***On*-site** **1**	**Zone I notch**	1	0	0	0	0	1
	**Zone I**	1	2	2	0	0	5
	**Post Zone II**	0	1	10	3	0	14
	**Zone II**	0	1	41	101	0	143
	**Zone III**	0	0	0	2	0	2
	**Total**	2	4	53	106	0	165
***On*-site** **2**	**Zone I notch**	1	0	0	0	0	1
	**Zone I**	0	0	2	0	0	2
	**Post Zone II**	0	0	6	9	0	15
	**Zone II**	0	0	3	126	0	129
	**Zone III**	0	0	0	3	0	3
	**Total**	1	0	11	138	0	150
***On*-site** **3**	**Zone I notch**	0	0	0	0	0	0
	**Zone I**	0	0	2	0	0	2
	**Post Zone II**	0	0	1	5	0	6
	**Zone II**	0	0	5	73	0	78
	**Zone III**	0	0	0	13	0	13
	**Total**	0	0	8	91	0	99
***On*-site** **all**	**Zone I notch**	2	0	0	0	0	2
	**Zone I**	1	2	6	0	0	9
	**Post Zone II**	0	1	17	17	0	35
	**Zone II**	0	1	49	300	0	350
	**Zone III**	0	0	0	18	0	18
	**Total**	3	4	72	335	0	414
	Agreement	Expected agreement		Kappa, wgt (95% CI)		SE	
*On*-site 1 × *off*-site	92.12%	87.69%		0.36 (0.26–0.46)		0.05	
*On*-site 2 × *off*-site	97.17%	93.99%		0.53 (0.41–0.59)		0.06	
*On*-site 3 × *off*-site	91.58%	90.00%		0.16 (0.10–0.36)		0.05	
ALL × *off*-site	94.32%	91.06%		**0.37 (0.37–0.40)**		0.04	

Note: Retinopathy of prematurity (ROP) zone tabulated between the *on*-site and *off*-site ophthalmologists for the right eyes. Abbreviations: weighted (wgt), confidence interval (CI), and standard error (SE).

**Table 7:** Level of agreement of the presence of plus disease—Right eye.

			*Off*-Site		
		**No Plus**	**Pre-Plus**	**Plus**	**Total**
***On*-site** **1**	**No plus**	134	11	0	145
	**Pre-plus**	3	16	0	19
	**Plus**	0	0	1	1
	**Total**	137	27	1	165
***On*-site** **2**	**No plus**	144	3	0	147
	**Pre-plus**	1	2	0	3
	**Plus**	0	0	0	0
	**Total**	145	5	0	150
***On*-site** **3**	**No plus**	82	2	0	84
	**Pre-plus**	4	11	0	15
	**Plus**	0	0	0	0
	**Total**	86	13	0	99
***On*-site** **all**	**No plus**	360	16	0	376
	**Pre-plus**	8	29	0	37
	**Plus**	0	0	1	1
	**Total**	368	45	1	414
	Agreement	Expected agreement	Kappa, wgt (95% CI)		SE
*On*-site 1 × *off*-site	95.76%	86.91%	0.68 (0.61–0.73)		0.07
*On*-site 2 × *off*-site	97.33%	94.80%	0.49 (0.06–0.92)		0.08
*On*-site 3 × *off*-site	93.94%	75.70%	0.75 (0.56–0.94)		0.10
ALL × *off*-site	94.20%	81.70%	**0.69** (**0.59–0.77**)		0.05

Note: Presence of plus disease evaluation tabulated between the *on*-site and *off*-site ophthalmologists for the right eyes. Abbreviations: weighted (wgt), confidence interval (CI), and standard error (SE).

## Data Availability

The fundus photographs generated and analyzed during the current study are not publicly available due to privacy concerns. As the images are identifiable and may reveal the identity of individual patients, they cannot be shared to protect patient confidentiality in accordance with ethical standards. The raw data supporting the conclusions of this article can be made available by the corresponding author on request.
